# Effect of Geometric Parameters on the Performance of P-Type Junctionless Lateral Gate Transistors

**DOI:** 10.1371/journal.pone.0095182

**Published:** 2014-04-17

**Authors:** Farhad Larki, Arash Dehzangi, Sawal Hamid Md Ali, Azman Jalar, Md. Shabiul Islam, Mohd Nizar Hamidon, Burhanuddin Yeop Majlis

**Affiliations:** 1 Institute of Microengineering and Nanoelectronics (IMEN), Universiti Kebangsaan Malaysia, Bangi, Selangor, Malaysia; 2 Functional Devices Laboratory, Institute of Advanced Technology, Universiti Putra Malaysia, Serdang, Selangor, Malaysia; Gazi University, Turkey

## Abstract

This paper examines the impact of two important geometrical parameters, namely the thickness and source/drain extensions on the performance of low doped p-type double lateral gate junctionless transistors (DGJLTs). The three dimensional Technology Computer-Aided Design simulation is implemented to calculate the characteristics of the devices with different thickness and source/drain extension and based on that, the parameters such as threshold voltage, transconductance and resistance in saturation region are analyzed. In addition, simulation results provide a physical explanation for the variation of device characteristics given by the variation of geometric parameters, mainly based on investigation of the electric field components and the carries density variation. It is shown that, the variation of the carrier density is the main factor which affects the characteristics of the device when the device's thickness is varied. However, the electric field is mainly responsible for variation of the characteristics when the source/drain extension is changed.

## Introduction

As the conventional planar metal-oxide-semiconductor field-effect transistors (MOSFETs) dimensions scale down to tens of nanometers, the formation of sharp source/channel and channel/drain junctions becomes crucial for suppressing the issues such as leakage current and short-channel effect (SCE). In nano scale devices, on the other hand, the formation of the ultra sharp junctions imposes severe challenges on doping techniques due to the difficulty to control the distribution of dopants at the metallurgical junctions and the intrinsic discreteness of the dopant itself. Recently, a novel device called junctionless transistor (JLT) has gain significant attention due to the advantages associated to the specific design of the device and simplification of the fabrication process [1], [2], [3]. From the structural point of view, the JLTs are heavily doped gated resistors with narrow silicon body in multiple gate architecture. Operationally, the JLTs are volume depleted in the *off* state (at zero gate bias) due to the effective work-function difference between the gate and the channel [4]. Conduction mechanism in JLTs only occurs in the bulk of the channel. Besides, no surface accumulation layer is formed below the threshold voltage [5]. The freedom of JLTs from different types of doping and associated gradients is considered as the main advantage of this design which simplifies the fabrication process. In addition, in subthreshold region device can obtain enhanced immunity from short channel effects in comparison to conventional inversion mode devices [6]. Performance comparison between bulk and SOI JLTs [7], [8], temperature dependence [9], scalability [10], [11], ballistic transport [12], and analog and digital applications of JLTs [13], [14] have been investigated in the literature.

Recently, the researchers of the present study have reported the fabrication and experimental characterization of low doped p-type junctionless transistors with single and double lateral gate(s) through the unconventional method of atomic force microscopy (AFM) nanolithography [15], [16], [17]. Simulations of carrier's transport and *pinch off* mechanism of the device have been presented in Refs. [18], [19]. Computational studies are absolutely critical to predict the ultimate performance of the nanoscale devices and to impel forward future experiments or technological developments. As a result, in this work, the impact of the two important geometric parameters to the operations of the p-type double lateral gate junctionless transistors (DGJLT) structure is comprehensively explored. First, the device simulation structures and models implemented in the present research are presented. Then, the operation and performance of the device in *on* and *off* state is briefly explained. Finally, the impact of geometric parameters of the device and particularly active layer thickness and source/drain extensions on characteristics of the device, are investigated based on the variation of hole density and electric field components.

## Methods

**Figure 1:**
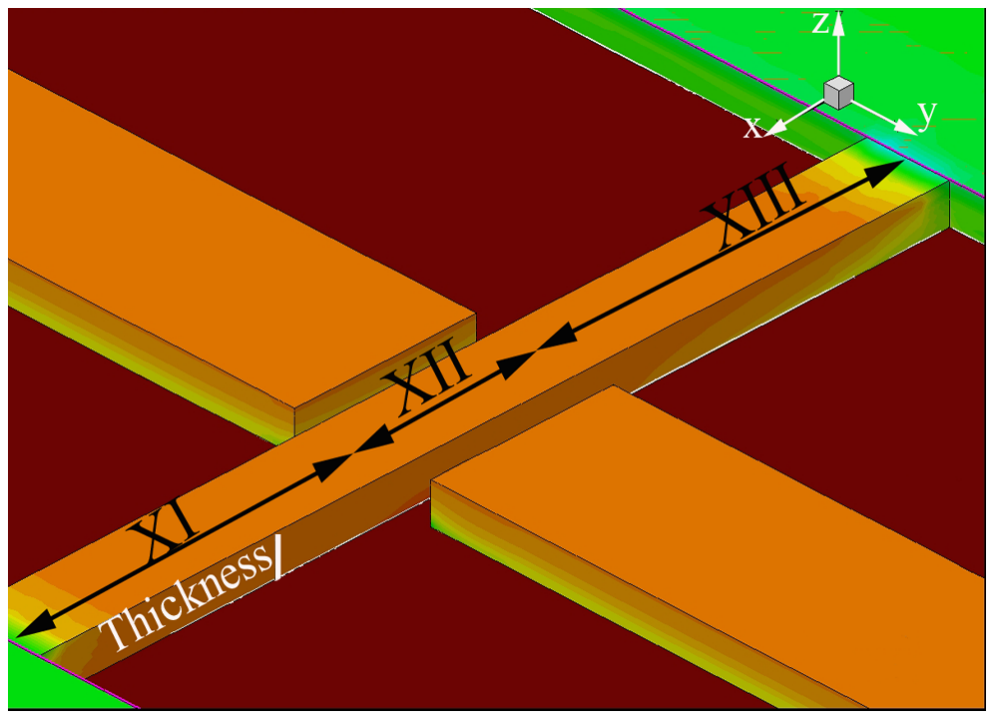
Isometric view of simulated DGJLT.

The structure of a typical DGJLT used for the simulations is shown in Fig. 1. Parameters used for the device simulations are listed in Table 1. The simulated structures have a uniform low doping concentration (Boron 10^15^) throughout all active regions of the device. The complete structure sits on a 145 nm ideal oxide. Different zones (X_I_, X_II_, and X_III_) correspond to the source extension, the channel and the drain extension, respectively are indicated for enhancement of the discussion and will be referred throughout the discussion.

**Table 1:** Simulated devices parameters.

Parameters	Value
Device layer thickness	20, 40, 100 nm
Zones X_I_ and X_III_ length	500 nm, 1 µm and 2 µm
Zone X_II_ length	200 nm
Contact work function	5.12 eV
Gate voltage	−2 V to +2 V
Drain voltage	−0.05 V to −1 V

The models used for the simulations are calibrated against a long-channel experimentally demonstrated DGJLT, as described in [18], [19]. We use a hydrodynamic model in Sentaurus software D-2010.03 [20] as the platform for the 3-D TCAD simulation presented in this study. Besides the fundamental equations, the doping-dependent Masetti mobility model and the default Shockley–Read–Hall (SRH) recombination-generation model is activated. SRH recombination-generation is applied in order to consider the leakage current and recombination through deep defect levels in the gap [21].

## Results and Discussion

### *On* and *off* state behavior of the device

**Figure 2:**
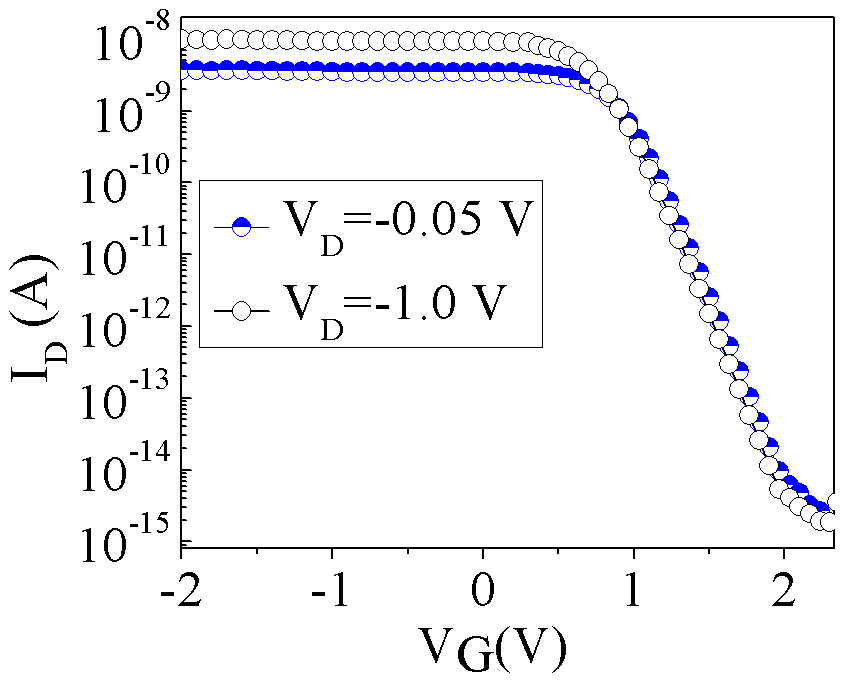
Transfer characteristics simulations of DGJLT with 100 µm width and thickness, nanowire length of 4.2μm, and channel length of 200 nm.

Fig. 2 shows the simulated transfer characteristics for the nominal DGJLT with 100 nm thickness and width for V_D_ = −0.05 V and −1.0 V. Zones X_I_, X_II_, and X_III_ have the doping density of 10^15^ cm^−3^, and the dimensions are considered as 2 µm, 200 nm, and 2 µm, respectively. The nominal simulated device is considered analogous to the fabricated device in order to give the basic view of the device operation. It can be seen that the device is in *on* state at zero gate voltage with *I_on_/I_off_* ratio of 10^7^. The device is driven into *off* state by increasing the positive gate voltage. However, negative gate voltage is not able to increase the current significantly. Since the device is in *on* state and already near the flatband condition at zero gate voltage, it can be claimed that the device is principally working similar to the *pinch-off* transistors [22], [23]. In the *on* state, the majority carriers moves in the volume of the channel and the depletion mechanism of the device due to the positive gate voltage applied to the lateral gates starts from the bottom corners of the channel at the Si/BOX interface, face to the lateral gates, and expands toward the center and top surface of the channel [18]. The electrostatic squeezing of the channel creates a large barrier along the holes transmission path. Increasing the drain voltage creates a depletion layer due to the sweeping of the carriers and at very high drain voltage, this depletion area along the electric field works as a barrier and prevents the electrical field from the propagating into the channel. This creates the current saturation, even for V_G_ = 0 V.

### Influence of thickness

**Figure 3:**
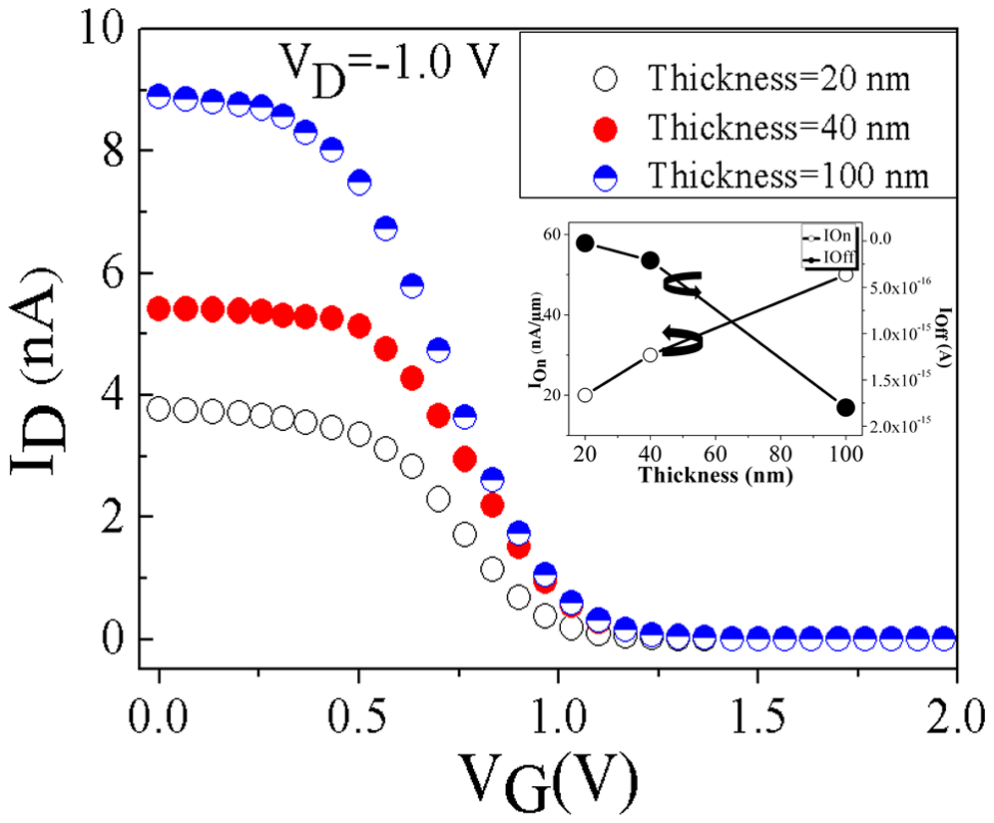
Transfer characteristics simulations of DGJLTs with three different thickness (20, 40, 100 nm). The inset plots the *on* (V_G_ = 0 V) and *off* (V_G_ = 2.0 V) current variation with thickness, at V_D_ = −1.0 V.

In the p-type DGJLTs, the conduction mechanism is through the current of majority carriers (holes) flowing in the bulk of the channel when the device is in the *on* state, and this volume as a conduction path would be gradually shrunk from bottom surface at Si/BOX interface to the top of the channel by increasing the positive gate voltage to turn the device *off*. This implies that the thickness has a strong influence on the characteristics and switch behavior of the DGJLTs. In order to investigation the effect of thickness on the behavior of the DGJLTs, the structures with thickness of 20 and 40 nm were simulated and the results compared to the nominal simulated device with 100 nm thickness. To avoid any quantum mechanical confinement effects, the thickness lower than 20 nm has not been investigated in this study. The dependence of transfer characteristics of DGJLT on thickness variation, when the drain is biased by V_D_ = −1.0 V is shown in Fig. 3. The inset shows the variation of *on* and *off* current with the thickness. As it is expected, the device with 20 nm thickness provides the highest *I_on_/I_off_* ratio (≈5×10^8^) and a small decrease of the *on* state drain current is obtained for thinner devices.

The threshold voltage variation over the change of the thickness (dV_th_/dT_Si_≈5 mV/nm) was negligible, mainly due to the suppression of random impurity fluctuation in these low doped devices [24]. The low doped channel in thin SOI devices has been implemented experimentally to minimize threshold voltage variations caused by random impurity fluctuation effects [25]. Another source of threshold voltage fluctuation is the scattering of source/drain doping atoms into the channel. This source of threshold voltage fluctuation is eliminated in DGJLTs because of the absence of junctions and doping gradients [26]. Based on the characteristics of the devices, we try to extract some of the important parameters in order to describe the variation of the device operation with respect to the thickness.

**Figure 4:**
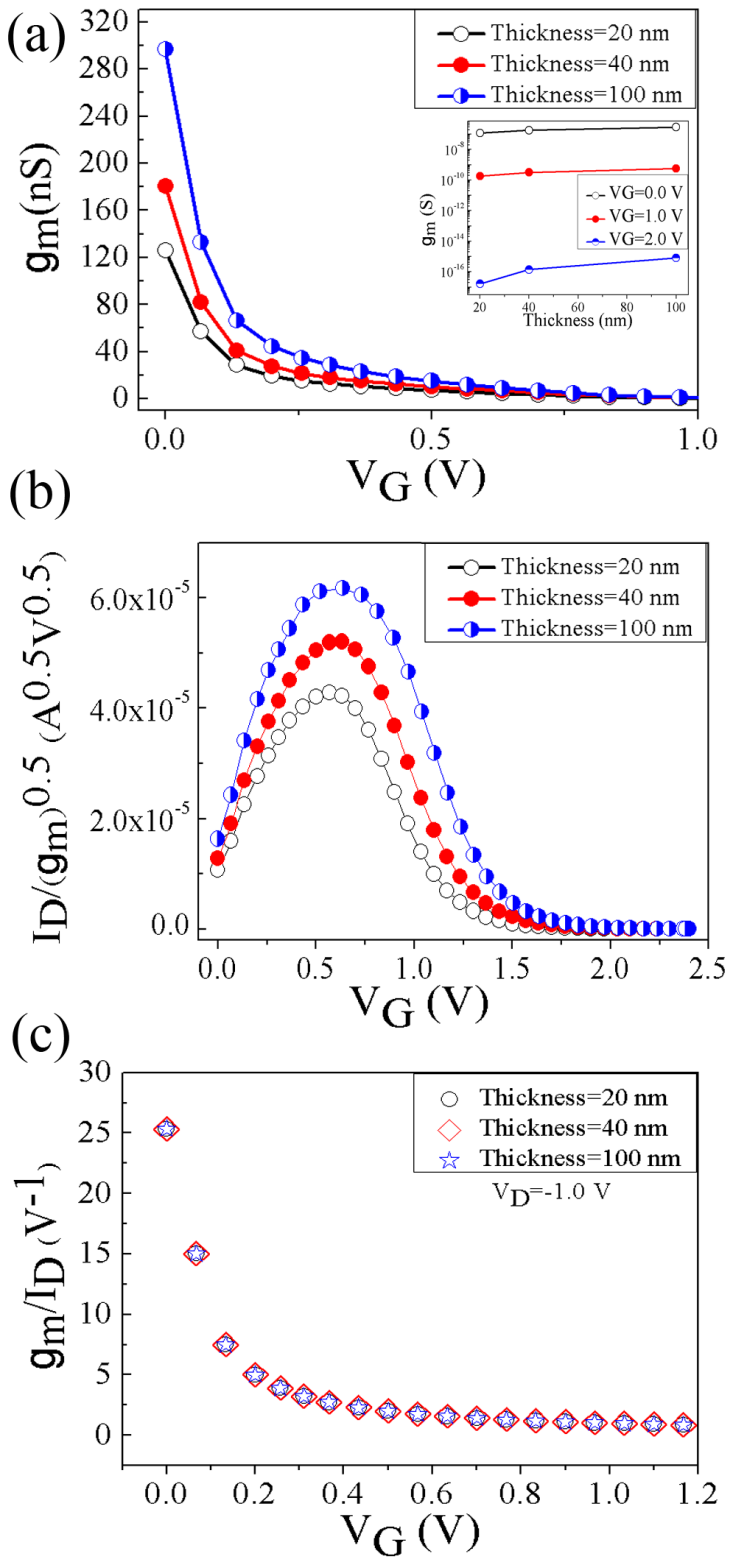
Variation of transconductance (g_m_) (a), plot of the ratio of the drain current to the square root of the transconductance (I_D_/g_m_^0.5^) (b) and g_m_/I_D_ versus gate voltage (c) for three different thicknesses (20, 40, and 100 nm) in saturation (V_D_ = −1.0 V).

The transconductance (g_m_) of the DGJLTs with three different thicknesses as a function of gate voltage is shown in Fig. 4a. The inset shows the variation of the g_m_ as a function of thickness at three different gate voltages. The overall trend demonstrates the variation of all devices from *on* to *off* state. Sharp decrease of transconductance shows the effect of positive gate voltage to deplete the channel according to the *pinch off* mechanism. Moreover, the lower transconductance in thinner device is attributed to the scarceness of the carriers flowing through the body of channel. Accordingly, the *on* state current is decreased in the thinner device.

A useful function for the extraction of the parameters in the conventional devices is the combination of the drain current and transconductance. This parameter (I_D_/g_m_^0.5^) known as Y(V_g_) is conventionally used to extract parameters such as, the threshold voltage and low field mobility [27]. The I_D_/g_m_^0.5^ variation versus gate voltage for devices with different thickness is plotted in Fig. 4b. Before investigating this function, it worth noting that, the operational principles of p-type DGJLTs, is different with that of gated resistors JLTs. In the gated resistor JLTs, below threshold the device is fully depleted. The conversion of the channel from full depletion which no conduction occurs to partial depletion which the bulk conduction starts can be achieved by increasing the gate voltage. Further increase in the gate voltage to the flatband voltage (V_fb_) makes channel to be completely neutral and above flatband an accumulation layer can be created at the interface with the gate [1], [5]. On the other hand, the DGJLTs are normally *on* device which forced to the *off* state by increasing the gate voltage and depleting the channel from the majority carriers. The variation of majority carriers density in the cross section of channel by increasing the gate voltage is shown in [18]. It is shown that, by small increasing of the gate voltage, the gates can just influence on the corner of the channel and it cannot affect the current significantly, mainly due to the lateral gates design and lack of the gate oxide. As a result, in I_D_/g_m_^0.5^ ratio, the numerator cannot be changed significantly; however the decrement of g_m_ in denominator is very sharp which is appeared as an increase of I_D_/g_m_^0.5^ in Fig. 4b. On the other hand, by increasing the gate voltage, the lateral gate influence on the current is more significant, while the transconductance variation is negligible. This behavior is shown with a graduate decrease in I_D_/g_m_^0.5^ for higher gate voltage. It should be noticed that, the lower value of I_D_/g_m_^0.5^ in thinner devices might be due the lower carrier concentration and lower value of transconductance; however in thinner devices the current increment is dominant.

A plot of g_m_/I_D_ as a function of gate voltage when the devices with different thickness are biased in the saturation region (V_D_ = −1.0 V), is shown in Fig. 4c. This ratio indeed is a universal characteristic of all the transistors belonging to a same process and known as a measure of the transconductance generation efficiency, since it is a measure of the efficiency of the device to transform current into transconductance [28]. When compared to the degradation of drive current and transconductance in thinner devices, the degradation in g_m_/I_D_ is seen to be smaller and g_m_/I_D_ is nearly identical for the different thickness. This trend is attributed to the fact that all devices have similar efficiency to transform current into transconductance. In addition, g_m_/I_D_ in JLTs is principally controlled by the body factor of the device which is equal to unity in these devices [14]. In the next section, in order to give a deeper physical perception about the effect of thickness variation on the device performance, we investigate the parameters which directly influence the output characteristics.

### Origins of characteristics change by thickness variation

In the DGLJTs, the gradient of carrier concentration along the source/drain and channel is zero, hence no diffusion can take place and the current is dominated by the drift current. For semiconductors with holes as majority carriers, the drift current under an applied field is given by [21]:
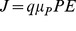
(1)hence the current can be written as
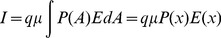
(2)where q is the electron charge, μ is mobility of carriers, P(x) is the carriers density (p(A)) integrated over the cross sectional area (A) of the device, and E is the electric field. It can be seen that, mobility, carrier density and electric field are factors which influence on output characteristics of the device, e.g. I_D_ and g_m_.

In the DGJLT the conduction path locates near the centre of the channel instead of being confined into the surface channel like conventional MOSFETs. This allows for the holes to move through the silicon with bulk mobility. In addition, due to the fabrication method based on the AFM nanolithography the surface and the body of the upper silicon layer in SOI remains intact. This makes the possibility to obtain more bulk properties, such as mobility value close to the bulk mobility even at very thin devices [15]. Accordingly, we can claim that the mobility variation is not the main factor for variation of characteristics of the device when thickness is varied. In this matter, as the most influential factors, the carrier density and electric field are taken into account for further investigation about thickness variation.

**Figure 5:**
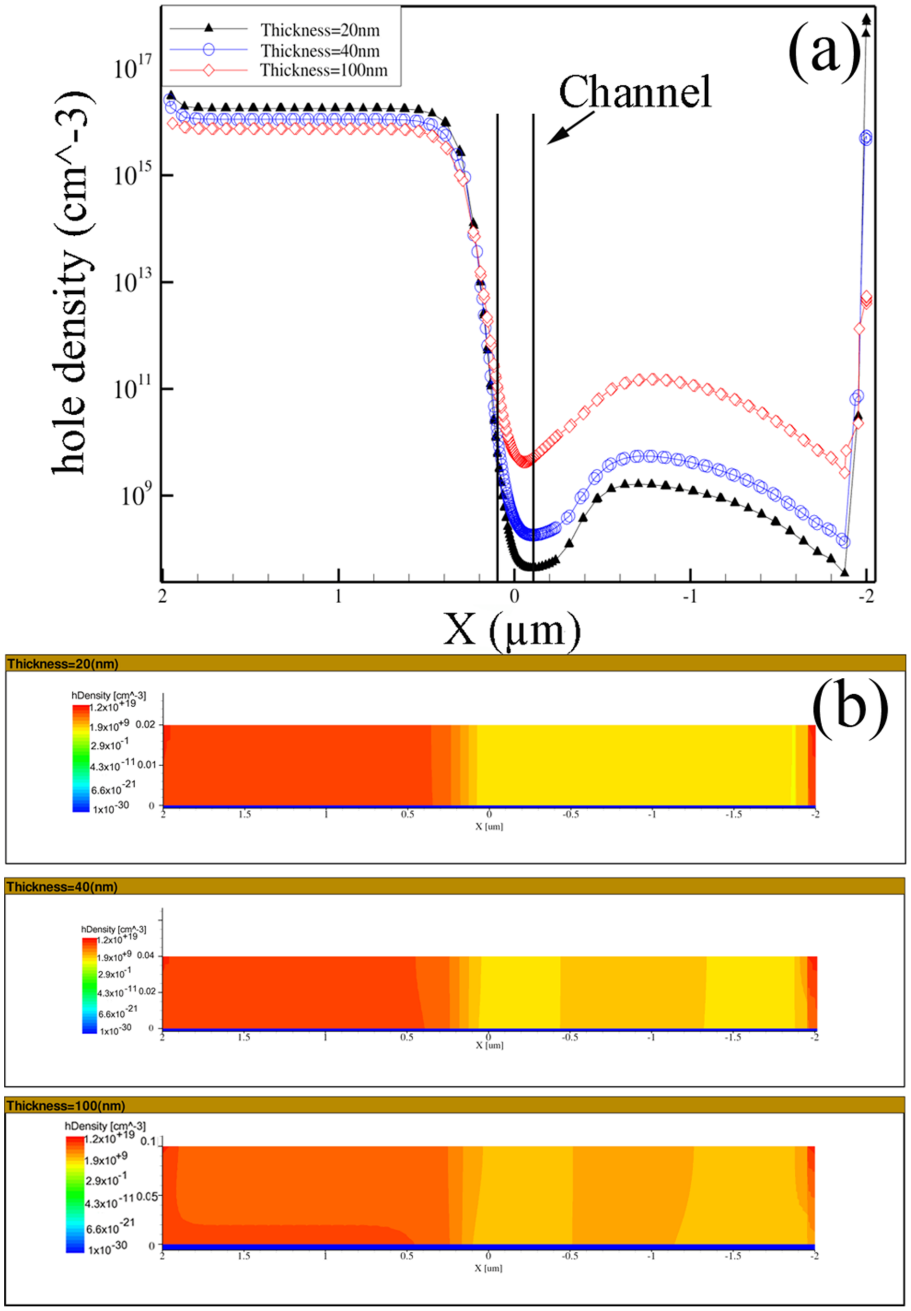
Hole density as a function of position along a horizontal cut line (a) and hole density along a vertical cut at y = 0 (b), for devices with different thicknesses of 20, 40, 100 nm. V_D_ = −1.0 V, V_G_ = +2 V.

The holes density (majority carriers) variation, along a longitudinal cutline and in a cross section of devices with three different thickness (20, 40, 100 nm) at V_G_ = +2 V and V_D_ = −1 V is presented in Fig. 5 (a–b), respectively. The effect of gate voltage variation on hole density for the nominal device was already investigated in Ref. [18]. In this gate voltage, the lateral gates have the highest impact on the channel with maximum variation of the carrier population. It is observed that, as the channel thickness decreased, the lateral gates were able to vary the carriers more effectively and consequently the current variation is more significant in the thinner devices. This trend confirms the variation of g_m_ results already presented in Fig. 4a, where lower g_m_ for smaller thickness can be explained by lower hole density in the channel.

**Figure 6:**
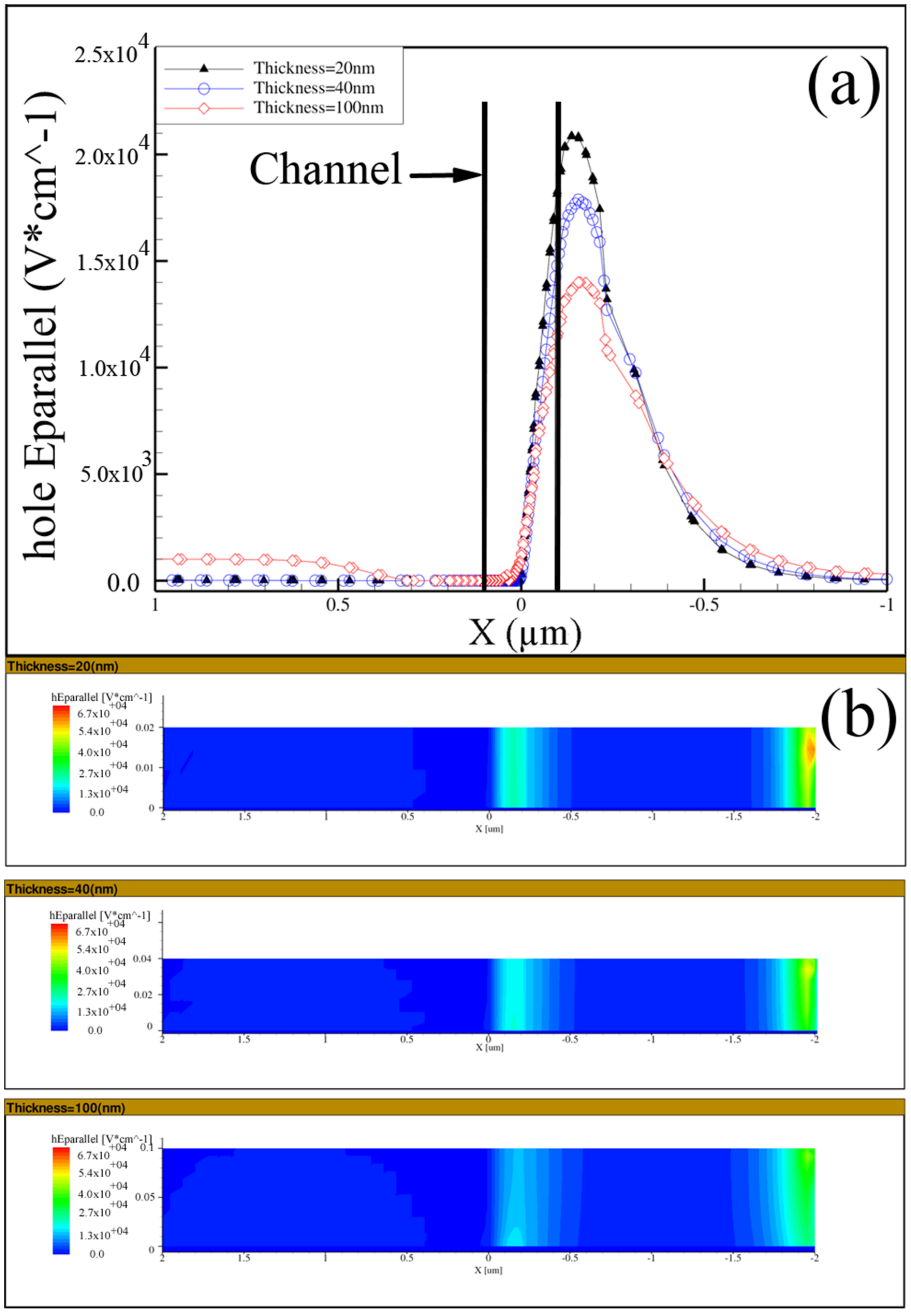
Electric field as a function of position along a horizontal cut line (a) and Electric field along a vertical cut at y = 0 (b), for devices with different thicknesses of 20, 40, 100 nm. V_D_ = −1.0 V, V_G_ = +2 V.

Figs. 6 (a–b) present the electric field along a horizontal line along the channel and in a cross section for devices with different thicknesses. It can be observed that, the peak of the electric field is occurred in the drain extension and not into the channel which confirms the typical behavior of the JLTs for all thicknesses [29]. The maximum electric field along the channel appeared in the device with the smallest thickness and for all devices the electric field reach to the lowest value at the centre of the channel (x = 0).

In fact, the overall electric field behavior along the nanowire was consistent with the hole density. Wherever the electric field was stronger the higher rate of hole depletion occurred. This trend for device with the lower thickness was even more intensified. The stronger electric field and the highest hole depletion were achieved at the lowest thickness (20 nm).

**Figure 7:**
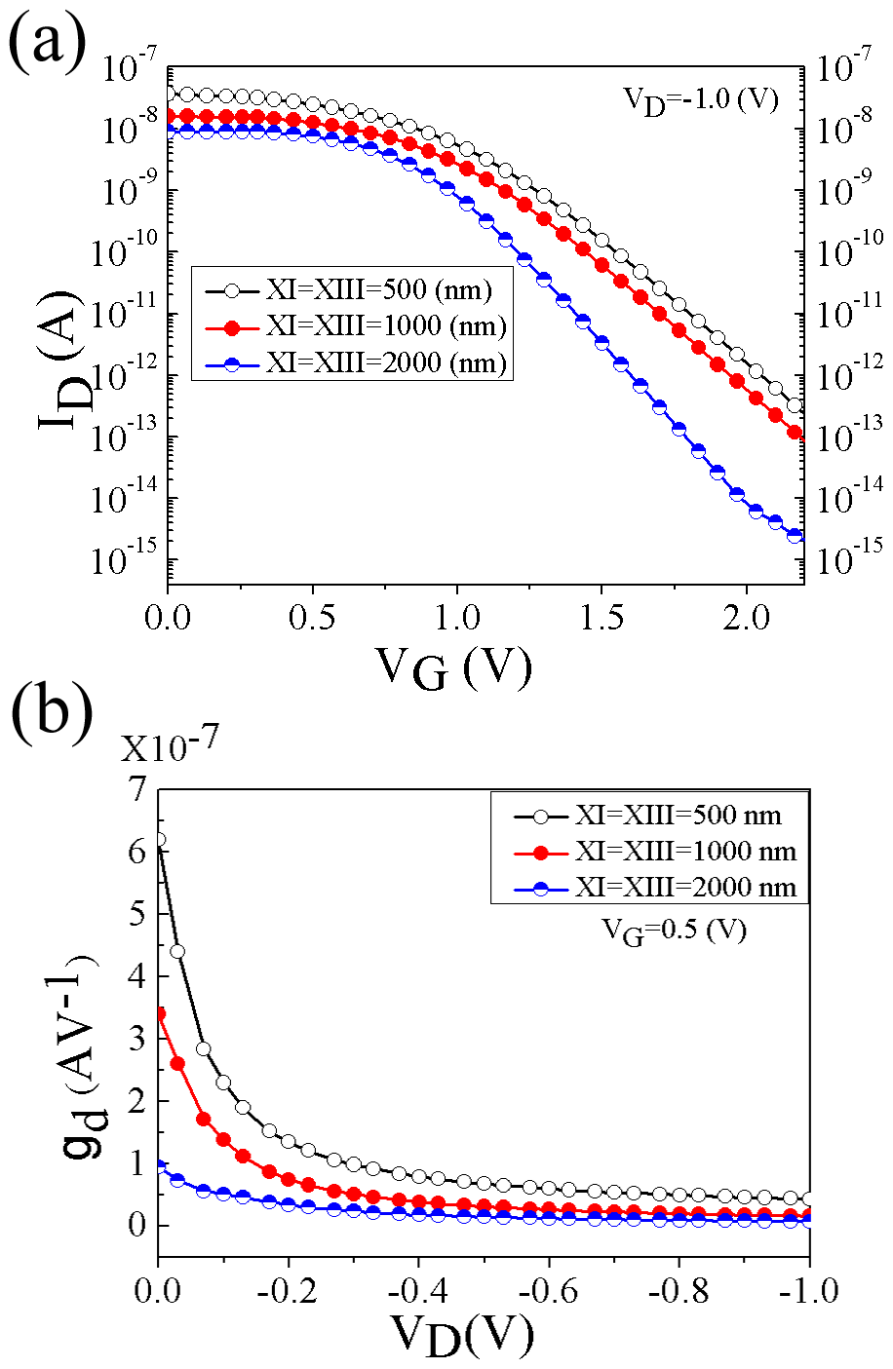
Transfer characteristics (I_D_–V_G_) (a) and drain conductance as a function of drain voltage (b), for different zones X_I_/X_III_ length at V_G_ = 0.5 V. Zones X_I_ and X_III_ have the length of 500, 1000, and 2000 nm. The length of zone X_II_ is constant at 200 nm.

According to the definition of the transconductance 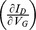 and considering that the value of the mobility is almost constant, from the differentiation of Equation. 2, we can obtain:
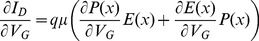
(3)This shows that two factors can directly cause the transconductance variation. The first factor is the variation of hole density with gate voltage multiplied by electric field along the channel (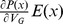) and the second factor is the variation of electric field with gate voltage multiplied by hole density along the channel (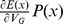). As it is presented in Figs 6a and 7a the hole density is significantly reduced with decreasing the thickness, however the magnitude of electric field is increased with decreasing the thickness. As the value of g_m_ is lowered with decreasing the thickness (Fig. 4a), then it can be concluded that (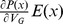) is the dominant factor contributing to the reduction of g_m_ in thinner devices.

### Influence of zones X_I_/X_III_ length, Fixed Zone X_II_

According to the specific design of the device, the variation of source/drain extension lengths (X_I_/X_III_) is another important geometric parameter which can significantly influence on the output characteristics. Since the nominal device was experimentally characterized with the channel length (X_II_) of 200 nm, the same X_II_ length is taken for all devices to purely investigate the effect of zones X_I_/X_III_ length variation. The transfer characteristics (I_D_–V_G_) and drain conductance variation as a function of drain voltage (g_d_–V_D_) of the devices with different zones X_I_/X_III_ length, in comparison with the nominal device, are presented in Figs. 7 (a–b). The device display typical characteristics over the investigated range of zones X_I_ and X_III_ lengths. *On* and *off* currents were increased by decreasing the zones X_I_ and X_III_ lengths. According to the model proposed in [30], in addition to the channel cross section, the output current in the flatband condition depends on the effective drain voltage at the channel (V_ch_) and doping concentration.

The transfer characteristics presented in Fig. 7aindicates that as the zones X_I_/X_III_ length scaled down to 500 nm, the subthreshold leakage increased by about 2 orders of magnitude resulting in degradation of *I_on_/I_off_* ratio. However, the comparison of drain conductance as a function of drain voltage for different zones X_I_/X_III_ length presented in Fig. 7b indicates that by decreasing zones X_I_/X_III_ length an increasing trend of the drain conductance is observed for all drain voltages. In order to understand the reason for increase of the *on* and *off* state current when the zones X_I_/X_III_ length decreases, some of the factors which influence on the characteristics of the device are analyzed.

**Figure 8:**
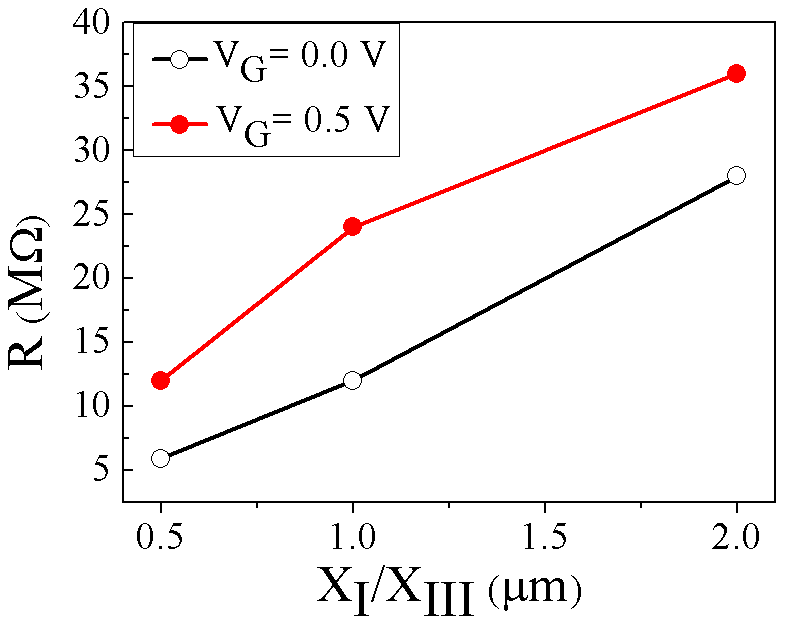
Total resistance versus zone X_I_/X_III_ length for two different gate voltages of 0.0 and 0.5 V, in the saturation region.

Total resistance (R_tot_) versus zones X_I_/X_III_ length obtained using the slope of the output characteristics data for devices with zones X_I_/X_III_ length of 0.5 µm, 1 µm, and 2 µm at two different gate voltages are shown in Fig. 8. The total resistance is varied from 6 MΩ for device with 0.5 µm X_I_/X_III_ length to 28 MΩ for device with 2 µm X_I_/X_III_ length, at V_G_ = 0 V. The resistance obtained includes the nanowire resistance (R_NW_), contacts resistance (R_c_) and the resistance of the external circuit (R_ext_) [31],
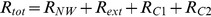
(4)The R_c1_ and R_c2_ could be estimated by simply extrapolating the curves to *L* = 0 since R_NW_ decreases to zero and *R*_tot_ asymptotically approaches the value of R_c_ [32]. By decreasing the zone X_I_/X_III_ the only part which varies is the R_NW_ due to the variation of length. Therefore, the lower R_NW_ in the device with 0.5 µm result in the enhanced *on* -state performance. However, with decreasing length the *off* state current (I*_off_*) is also increased, which is not favorable. The reason for I*_off_* value increment can be understood by investigating the electric field and hole density variation along the channel.

**Figure 9:**
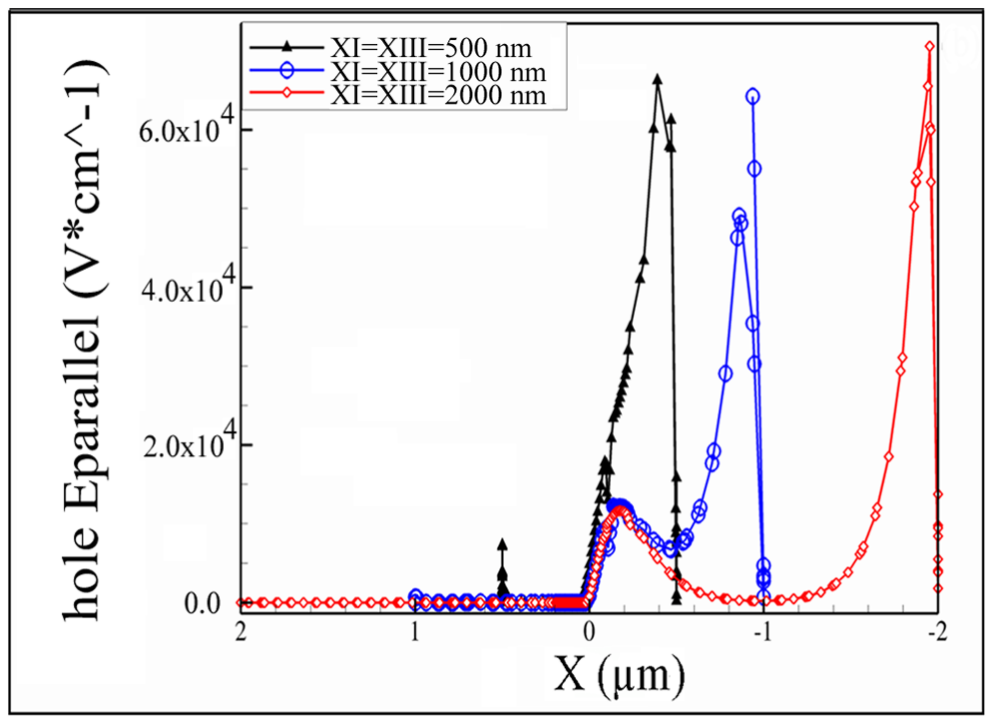
Electric field along the horizontal cut line for different zones X_I_ and X_III_ lengths, V_D_ = −1.0 V, V_G_ = +2 V.

The electric field as a function of position along the current direction in devices with three different zones X_I_/X_III_ lengths is presented in Fig. 9. It is shown that, the interaction of electric fields from lateral gates and drain contact was diminished in the channel and enhanced in drain extension for all devices. However, for the device with the shortest length (X_I_ = X_III_ = 500 nm), the electric field was stronger at the drain extension compared to the devices with longer X_I_/X_III_ length. Moreover, the peak of the electric field is located closer to the channel. For the short X_I_/X_III_ length, the strong electric field from the drain could overcome the zone X_III_ and suck the holes directly from the channel or even zone X_I_ (punching effect). The carriers which are dislocated by electric field need to be relocated somewhere in the channel (zone X_II_) or in the zone X_III_. This can vary the carrier configuration in different parts of the device.

**Figure 10:**
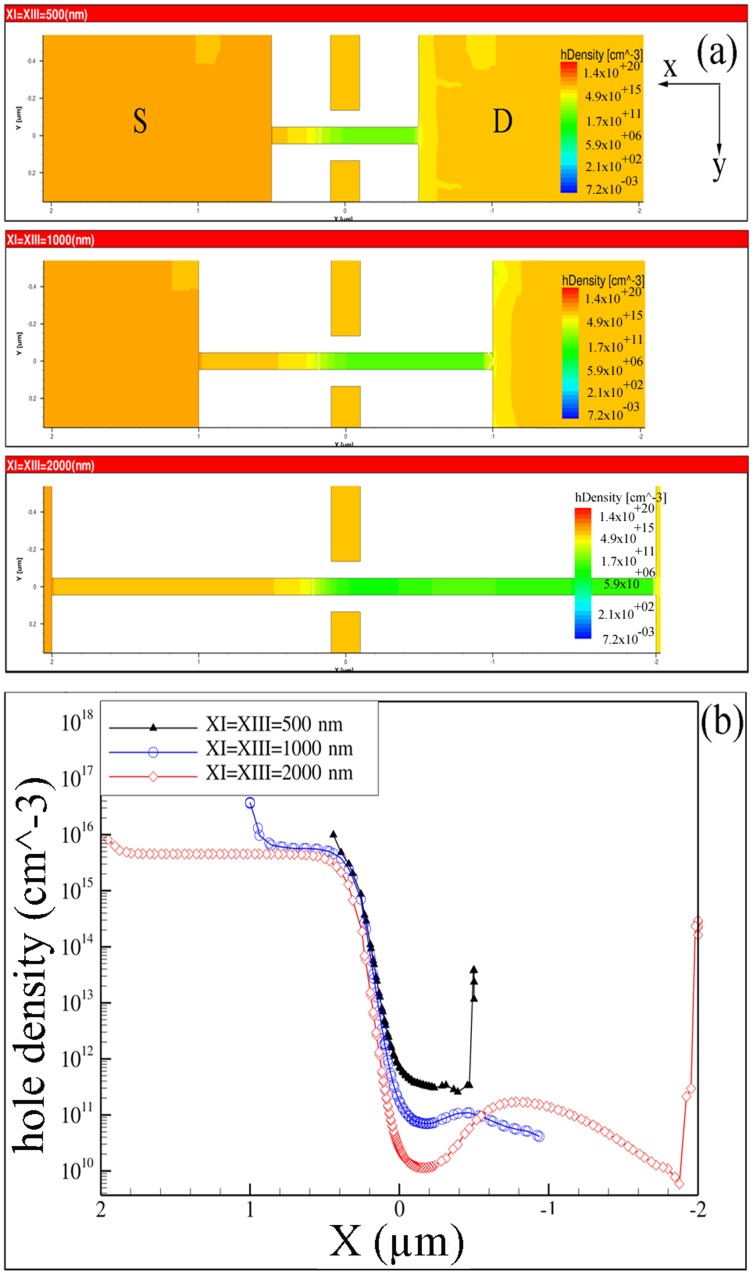
Simulated hole density as a function of position along a cut at Z = 50 nm (a) and hole density as a function of position along a horizontal cut line (b), for different zones X_I_ and X_III_ lengths at V_D_ = −1.0 V and V_G_ = +2 V.

The holes density variation along the cut at Z = 50 nm and along a cutline as a function of position for the devices with three different zones X_I_/X_III_ length are shown in Fig. 10 (a–b), respectively. It can be observed that the lowest depletion in the channel occurred for the shortest length (X_I_ = X_III_ = 500 nm). For the devices with longer length, the depleted carriers from channel could be spread into the zone X_III_.

However, for the device with the shortest length, there is no area for the depleted holes to be located because of the punching effect and drain influence. This could provide the lower rate of depletion in the channel at *off* state and explain the higher subthreshold current for the device with the shortest length.

### Conclusion

Thickness and source/drain extension lengths as two important geometric parameters of the double lateral gate junctionless transistors (DGJLT) have been investigated using 3-D TCAD simulation tools. The device has shown a good scaling ability in the investigated parameters. Unlike the high doped JLTs, the threshold voltage remains approximately insensitive to the variation of investigated parameter. The subthreshold leakage current shows strong sensitivity to both thickness and zone X_I_/X_III_ length variation. It is concluded that, when the thickness of the device is scaled down the carrier's density variation is the main factor which influence the output characteristics of the device. However, for further scaling the zone X_I_/X_III_ length the electric fields penetration inside the channel is the main source of increases in leakage current. The dependency of the devices' characteristics to the scaling of the investigated parameters is summarized in Table 2. These modeling results clearly encourage what our previous experimental work has proposed: that the junctionless lateral gate transistor can be used to construct extremely simple transistor device.

**Table 2:** Dependency of the devices' characteristics to the scaling of thickness and zones X_I_/X_III_ length.

	Thickness scaling	Zone X_I_/X_III_ scaling
**Threshold voltage (V_th_)**	≈Insensitive	≈Insensitive
**Leakage current**	Decreases	Increases
**Hole density in the channel**	Decreases	Increases
**Electric field**	Increases	Increases
**The most Influential factor**	Hole density	Electric field
